# Supplementary material for: Subcellular localization of type IV pili regulates bacterial multicellular development
Source: Nat Commun. 2022 Oct 25;13:6334. doi: 10.1038/s41467-022-33564-7 (PMC9596432; doi:10.1038/s41467-022-33564-7)
Supplement: Supplementary file 1 — Supplementary Information [file 41467_2022_33564_MOESM1_ESM.pdf]

# Supplementary Information

## Supplementary References


1. Ellison, C. K. *et al.* Acinetobacter baylyi regulates type IV pilus synthesis by employing two extension motors and a motor protein inhibitor. *Nat Commun* **12**, 3744 (2021).

2. Jacobs, M. A. et al. Comprehensive transposon mutant library of *Pseudomonas aeruginosa*. *Proceedings of the National Academy of Sciences of the United States of America* **100**, 14339–14344 (2003).

3. Bertrand, J. J., West, J. T. & Engel, J. N. Genetic analysis of the regulation of type IV pilus function by the Chp chemosensory system of Pseudomonas aeruginosa. *Journal of Bacteriology* **192**, 994–1010 (2010).

4. Sievers, F. et al. Fast, scalable generation of high-quality protein multiple sequence alignments using Clustal Omega. *Molecular Systems Biology* **7**, 539 (2011).


**Supplementary Table 1**. Bacterial strains used in this study.

| **Strain CE#** | **Strain ZG#** | **Strain name in manuscript** | **Genotype** | **Figures where strain appears†** | **Ref** |
|---|---|---|---|---|---|
| CE100 | ZG1706 | Parent | *A. baylyi* ADP1 *comP*$^{T129C}$ | 1A, 2C, S3B, S7 | 1 |
| CE317 | ZG1707 | Δ*pilT* or Parent | ADP1 *comP*$^{T129C}$ Δ*pilT*::*spec* | 1C, 1D, 2D, 4B, 4C, S7, S10 | 1 |
| CE6 | ZG1668 | Δ*pilG* Δ*pilT* | ADP1 *comP*$^{T129C}$ Δ*pilG* Δ*pilT*::*kan* | 1C, 1D, 2D | This study |
| CE7 | ZG1669 | Δ*pilH* Δ*pilT* | ADP1 *comP*$^{T129C}$ Δ*pilH* Δ*pilT*::*kan* | 1C, 1D, 2D | This study |
| CE18 | ZG1670 | Δ*pilI* Δ*pilT* | ADP1 *comP*$^{T129C}$ Δ*pilI* Δ*pilT*::*kan* | 1C, 1D, 2D | This study |
| CE19 | ZG1671 | Δ*pilJ* Δ*pilT* | ADP1 *comP*$^{T129C}$ Δ*pilJ* Δ*pilT*::*kan* | 1C, 1D, 2D | This study |
| CE20 | ZG1672 | Δ*chpA* Δ*pilT* | ADP1 *comP*$^{T129C}$ Δ*chpA* Δ*pilT*::*kan* | 1C, 1D, 2D | This study |
| CE315 | ZG1676 | Δ*fimL* Δ*pilT* or Δ*fimL* | ADP1 *comP*$^{T129C}$ Δ*fimL*::*kan* Δ*pilT*::*spec* | 1C, 1D, 2D, S6A | This study |
| CE256 | ZG1681 | PilQ-mCherry or Parent | ADP1 *comP*$^{T129C}$ *pilQ*::*pilQ-mCherry, gent,* $P_{tac}$- *aroK* | 2A, S3B | This study |
| CE331 | ZG1682 | PilQ-GmR | ADP1 *comP*$^{T129C}$ *pilQ*::*pilQ, gent,* $P_{tac}$-*aroK* | S3B | This study |
| CE66 | ZG1679 | Δ*pilQ* | ADP1 *comP*$^{T129C}$ Δ*pilQ* | S3B | 1 |
| CE320 | ZG1700 | Δ*pilG* | ADP1 *comP*$^{T129C}$ *pilQ*::*pilQ-mCherry, gent,* $P_{tac}$- *aroK* Δ*pilG* | 2B | This study |
| CE321 | ZG1701 | Δ*pilH* | ADP1 *comP*$^{T129C}$ *pilQ*::*pilQ-mCherry, gent,* $P_{tac}$- *aroK* Δ*pilH* | 2B | This study |
| CE322 | CE1702 | Δ*pilI* | ADP1 *comP*$^{T129C}$ *pilQ*::*pilQ-mCherry, gent,* $P_{tac}$- *aroK* Δ*pilI* | 2B | This study |

| | | | | | |
|---|---|---|---|---|---|
| CE323 | ZG1703 | Δ*pilJ* | ADP1 *comP*$^{T129C}$ *pilQ*::*pilQ-mCherry, gent,* $P_{tac}$- *aroK* Δ*pilJ* | 2B | This study |
| CE324 | ZG1704 | Δ*chpA* | ADP1 *comP*$^{T129C}$ *pilQ*::*pilQ-mCherry, gent,* $P_{tac}$- *aroK* Δ*chpA* | 2B | This study |
| CE328 | ZG1708 | Δ*fimL* | ADP1 *comP*$^{T129C}$ *pilQ*::*pilQ-mCherry, gent,* $P_{tac}$- *aroK* Δ*fimL*::*kan* | 2B | This study |
| CE2 | ZG1693 | Δ*pilG* | ADP1 *comP*$^{T129C}$ Δ*pilG* | 2C | This study |
| CE3 | ZG1694 | Δ*pilH* | ADP1 *comP*$^{T129C}$ Δ*pilH* | 2C | This study |
| CE8 | ZG1695 | Δ*pilI* | ADP1 *comP*$^{T129C}$ Δ*pilI* | 2C | This study |
| CE9 | ZG1696 | Δ*pilJ* | ADP1 *comP*$^{T129C}$ Δ*pilJ* | 2C | This study |
| CE10 | ZG1697 | Δ*chpA* | ADP1 *comP*$^{T129C}$ Δ*chpA* | 2C | This study |
| CE233 | ZG1699 | Δ*fimL* | ADP1 *comP*$^{T129C}$ Δ*fimL*::*kan* | 2C | This study |
| CE358 | ZG1812 | Δ*comP* | ADP1 Δ*comP*::*kan* | 2C | TND0114[1] |
| CE250 | ZG1698 | *fimL*-3xFLAG | ADP1 *comP*$^{T129C}$ *fimL*::*fimL-3xFLAG* | | This study |
| CE724 | ZG1795 | Δ*fimV* Δ*pilT* or Δ*fimV* | ADP1 *comP*$^{T129C}$ Δ*fimV*::*chlor* Δ*pilT*::*spec* | 3A, 4B, 4C, S10 | This study |
| CE17 | ZG1686 | PilG-mRuby3 Parent | ADP1 *comP*$^{T129C}$ *pilG*::*pilG-mRuby3* | 3B, 3C | This study |
| CE257 | ZG1689 | PilG-mRuby3 Δ*fimL* | ADP1 *comP*$^{T129C}$ *pilG*::*pilG-mRuby3* Δ*fimL*::*chlor* | 3B, 3C | This study |
| CE681 | ZG1796 | PilG-mRuby3 Δ*fimV* | ADP1 *comP*$^{T129C}$ *pilG*::*pilG-mRuby3* Δ*fimV*::*kan* | 3B, 3C, S6B | This study |
| CE39 | ZG1797 | PilG-mRuby3 Δ*comP* | ADP1 *pilG*::*pilG-mRuby3* Δ*comP*::*kan* | 3B, 3C | This study |
| CE251 | ZG1798 | FimL-mCherry Parent | ADP1 *comP*$^{T129C}$ *fimL*::*fimL-mCherry* | 3B, 3C | This study |
| CE258 | ZG1799 | FimL-mCherry Δ*pilG* | ADP1 *comP*$^{T129C}$ *fimL*::*fimL-mCherry* Δ*pilG* | 3B, 3C | This study |
| CE682 | ZG1800 | FimL-mCherry Δ*fimV* | ADP1 *comP*$^{T129C}$ *fimL*::*fimL-mCherry* Δ*fimV*::*kan* | 3B, 3C | This study |
| CE254 | ZG1801 | FimL-mCherry Δ*comP* | ADP1 *fimL*::*fimL-mCherry* Δ*comP*::*spec* | 3B, 3C | This study |
| CE745 | ZG1802 | FimV-mRuby3 Parent | ADP1 *comP*$^{T129C}$ *fimV*::*fimV-mRuby3* | 3B, 3C | This study |
| CE757 | ZG1803 | FimV-mRuby3 Δ*pilG* | ADP1 *comP*$^{T129C}$ *fimV*::*fimV-mRuby3* Δ*pilG* | 3B, 3C, S6C | This study |
| CE760 | ZG1804 | FimV-mRuby3 Δ*fimL* | ADP1 *comP*$^{T129C}$ *fimV*::*fimV-mRuby3* Δ*fimL::chlor* | 3B, 3C | This study |
| CE919 | ZG1805 | FimV-mRuby3 Δ*comP* | ADP1 *fimV*::*fimV-mRuby3* Δ*comP*::*spec* | 3B, 3C | This study |
| CE25 | ZG1736 | PilG-mRuby3 Δ*pilT* | ADP1 *comP*$^{T129C}$ *pilG*::*pilG-mRuby3* Δ*pilT*::*kan* | S5 | This study |
| CE819 | ZG1806 | FimL-mCherry Δ*pilT* | ADP1 *comP*$^{T129C}$ *fimL*::*fimL-mCherry* Δ*pilT*::*spec* | S5 | This study |
| CE820 | ZG1807 | FimV-mRuby3 Δ*pilT* | ADP1 *comP*$^{T129C}$ *fimV*::*fimV-mRuby3* Δ*pilT*::*spec* | S5 | This study |

| | | | | | |
|---|---|---|---|---|---|
| CE719 | ZG1813 | Δ*fimL* + *fimL* | ADP1 *comP*$^{T129C}$ Δ*fimL*::*chlor* Δ*pilT*::*spec* Δ*vanAB*::*kan*, $P_{tac}$-*fimL* | S6A | This study |
| CE1017 | ZG1814 | FimV-mRuby3 Δ*pilG* + *fimL* | ADP1 *comP*$^{T129C}$ *fimV*::*fimV-mRuby3* Δ*pilG* Δ*vanAB*::*kan*, $P_{tac}$-*fimL* | S6C | This study |
| CE1021 | ZG1815 | PilG-mRuby3 Δ*fimV* + *fimL* | ADP1 *comP*$^{T129C}$ *pilG*::*pilG-mRuby3* Δ*fimV::chlor* Δ*vanAB*::*kan*, $P_{tac}$-*fimL* | S6B | This study |
| CE851 | ZG1808 | Δ*comP* | ADP1 Δ*comP*::*spec* Δ*pilT*::*kan* | 4B, 4C, S10 | This study |
| CE1000 | ZG1809 | Δ*fimV* Δ*comP* | ADP1 Δ*fimV*::*chlor* Δ*comP*::*spec* Δ*pilT*::*kan* | 4B, 4C, S10 | This study |
| CE1012 | ZG1810 | Parent | ADP1 *comP*$^{T129C}$ Δ*pilT*::*kan* Δ*vanAB*::*apr*, $P_{tac}$-*mRuby3* | 4D | This study |
| CE1013 | ZG1811 | Δ*fimV* | ADP1 *comP*$^{T129C}$ Δ*fimV*::*chlor* Δ*pilT*::*kan* Δ*vanAB*::*apr*, $P_{tac}$-*mRuby3* | 4D | This study |
| CE115 | ZG500 | Parent | *Pseudomonas aeruginosa* PAO1 | S7 | 2 |
| CE1189 | ZG1183 | Δ*pilTU* | *P. aeruginosa* PAO1 Δ*pilTU* | S7 | 3 |

*Send strain requests to CKE; † Supplementary figures referred to as "S#"

**Supplementary Table 2**. Primers used for strain construction.

| Primer name | Primer sequence 5' → 3' (overlapping regions underlined, point mutations or 3xFLAG tag in bold) | Description |
|---|---|---|
| ABD123 | ATTCCGGGGATCCGTCGAC | AbR cassette F |
| ABD124 | TGTAGGCTGGAGCTGCTTC | AbR cassette R |
| CE49 | GTACTCATCTCGCATATTCAGGAAATG | ΔpilTF1 |
| CE50 | CGCAAGTTCAACAGTCCTACCAA | ΔpilTR1 |
| CE31 | GCTGTTTGATTTTATCCAGAACCTTG | ΔpilG F1 |
| CE61 | TGACGTGATTACGAATTGCGGTGATTTTGATCACTTTGGATTTGTCTTCC | ΔpilG R1 |
| CE62 | GGAAGACAAATCCAAAGTGATCAAAATCACCGCAATTCGTAATCACGTCA | ΔpilG F2 |
| CE34 | GCGCTGTAATTACAATAGTGTTCGGA | ΔpilG R2 and pilG-mRuby3 R2 |
| CE37 | GGTGGTACGCAGTTGTGGTAAAAT | ΔpilH F1 and pilG-mRuby3 F1 |
| CE63 | ATCATCCGTATGAATATATTGCTGGATTGAATCATCAACAATCAAAATACGTGCCA | ΔpilH R1 |
| CE64 | TGGCACGTATTTTGATTGTTGATGATTCAATCCAGCAATATATTCATACGGATGAT | ΔpilH F2 |
| CE40 | GGCAACTCAATTCAAACTGATGTACTT | ΔpilH R2 |
| CE101 | TTCAACGCTCATTCGTTGCCAT | ΔpilI F1 |
| CE102 | ATCAATATTGTGAAGCATTCATAAAATCTGTCAAACCAGCAGATGTGGTTGC | ΔpilI R1 |
| CE103 | GCAACCACATCTGCTGGTTTGACAGATTTTATGAATGCTTCACAATATTGAT | ΔpilI F2 |
| CE104 | CAGTTGACTGGTTACTGGTTCTGC | ΔpilI R2 |
| CE107 | GGTTTTTCCAGTTTTTAATGCTGCC | ΔpilJ F1 |
| CE108 | TCAGATGCAAAATCAAGCTTATACGTCGCTTTATTTTTTTCCGATTGATTGA | ΔpilJ R1 |
| CE109 | TCAATCAATCGGAAAAAAATAAAGCGACGTATAAGCTTGATTTTGCATCTGA | ΔpilJ F2 |
| CE110 | CCGAACAGTACGCTGCAATC | ΔpilJ R2 |
| CE29 | AATATCTTGTCGCCGATTCATTGAAA | ΔchpA F1 |
| CE113 | GGTTTTGGCTATGTTTAATCGCTTTCGTCTCTATAAGATGATTTACTTGTGCA | ΔchpA R1 |

| | | |
|---|---|---|
| CE114 | TGCACAAGTAAATCATCTTATAGAGACGAAAGCGATTAAACATAGCCAAAACC | ΔchpA F2 |
| CE115 | CTGTATTTTGATGCAATGTTGGAGATT | ΔchpA R2 |
| CE735 | GTGCACTGGGGATATCTGCACT | ΔfimL F1 |
| CE736 | GTCGACGGATCCCCGGAATATCGAAGCGAATAGAAACTTGTGAAGAC | ΔfimL R1 |
| CE737 | GAAGCAGCTCCAGCCTACAAACGCCTCCCAATTTCAATCAACG | ΔfimL F2 |
| CE738 | TGCTAACAGAATTGTATGTGTCTACGCC | ΔfimL R2 and fimL-3xFLAG R2 and fimL-mCherry R2 |
| CE790 | **ATCATGATCTTTATAATCACCATCATGATCTTTATAATC**GGCAACCGTTGATTGAAATTGGGAG | fimL-3xFLAG R1 |
| CE791 | **GATGGTGATTATAAAGATCATGATATTGATTATAAAGATGATGATGATAAA**TCCCAATTTCAATCAACGGTTGCC | fimL-3xFLAG F2 |
| CE1525 | GCCCCGTAATAGTAATTCAAAGTACATGG | ΔfimV F1 |
| CE1526 | GTCGACGGATCCCCGGAATTCGTATTATAAAAATGATCGCCACGACC | ΔfimV R1 |
| CE1527 | GAAGCAGCTCCAGCCTACAAGCGCTCAACAGCTTGAACTC | ΔfimV F2 |
| CE1528 | GTATAAATGAAGAACATACAAGGAAGGGCG | ΔfimV R2 and fimV-mRuby3 R2 |
| CE590 | ATGAGAACAGATTTTAACGTTTTTACGACTGG | PilQ-mCherry up F |
| CE196 | GAACCAGCAGCTGAACCAGCTGAACCATGATTTCCAACAATAGTGTCATTAACTATTCG | PilQ-mCherry up R |
| CE197 | TTCAGCTGCTGGTTCAGGTGAATTTATGGTTTCCAAGGGCGAGGA | PilQ-mCherry mCherry region F |
| CE198 | TTACTTGAATTAAAACTATATTATTTGTACAGCTCATCCATGCCA | PilQ-mCherry mCherry region R |
| CE637 | TATAGTTTTAATTCAAGTAATTCAATAGGTGACTCCATTCCGGGGATCCGTCGAC | PilQ-mCherry AbR region F1 |
| CE593 | TCGTTGCTGCTGCGTAACATGCGAAACTATCCTCATCCTGTCTCTTG | PilQ-mCherry AbR region R1 |
| CE594 | CAGGATGAGGATAGTTTCGCATGTTACGCAGCAGCAACGATG | PilQ-mCherry AbR region F2 |
| CE595 | GGAACTTCAAGATCCCCTTATTAGGTGGCGGTACTTGGGTC | PilQ-mCherry GmR region R2 |
| CE596 | GACCCAAGTACCGCCACCTAATAAGGGGATCTTGAAGTTCCTATTCCG | PilQ-mCherry Ptac region F |
| CE336 | GCTTAATTACCTCCTAATTGAATTCCTAGGC | PilQ-mCherry Ptac region R |
| CE638 | GGAATTCAATTAGGAGGTAATTAAGCTTGCCAAGCAAAGAGTTTGACACC | PilQ-mCherry down F |
| CE592 | TCTAATGCCGCTGCACTGTCAC | PilQ-mCherry down R |
| CE146 | TTCACCTGAACCAGCAGCTGAACCAGCTGAACCTACACTGACGTGATTACGAATTGCG | pilG-mRuby3 R1 |
| CE149 | GGGTGGAATGGATGAATTATATAAGTAACACGATATTGTTTTGAGGTCTTTATGGCAC | pilG-mRuby3 F2 |
| CE147 | TGGTTCAGCTGCTGGTTCAGGTGAATTTATGGTATCTAAGGGTGAAGAATTGATTAAGG | mRuby3 F |
| CE148 | TTACTTATATAATTCATCCATTCCACCCCCAAG | mRuby3 R |
| CE748 | ATGTGGTGGTGAAGCAGAAACG | fimL-3xFLAG F1 and fimL-mCherry F1 |
| CE749 | CACCTGAACCAGCAGCTGAACCAGCTGAACCGGCAACCGTTGATTGAAATTGGGAG | fimL-mCherry R1 |
| CE751 | GCATGGATGAGCTGTACAAATCCCAATTTCAATCAACGGTTGCC | fimL-mCherry F2 |

| | | |
|---|---|---|
| CE197 | TTCAGCTGCTGGTTCAGGTGAATTTATGGTTTCCAAGGGCGAGGA | mCherry F |
| CE750 | ACCGTTGATTGAAATTGGGATTTGTACAGCTCATCCATGCCACC | mCherry R |
| CE1683 | AACCGCAACCTGATAAGGATAATCC | fimV-mRuby3 F1 |
| CE1717 | TCCACCACTTCCACCTGCAGATGCCATTCGATTTAGCAGTTTTTTTG | fimV-mRuby3 universal linker R1 |
| CE1718 | GCAGGTGGAGCAGGTGGATAAAAAATATCGTTCTAACATAAGCAGTCAGTTTG | fimV-mRuby3 universal linker F2 |
| CE1555 | GCAGGTGGAAGTGGTGGA | Universal linker mRuby3 F |
| CE1556 | TCCACCTGCTCCACCTGC | Universal linker mRuby3 R |
| CE317 | GCAAACCACAAACATAATGTTTGAAATCC | vanAB F1 for ectopic expression constructs |
| CE406 | GAAGCAGCTCCAGCCTACA | vanAB F2 for ectopic expression constructs |
| CE176 | CCAAGACTATAAATAATCGACATGATCAATTTTAA | vanAB R2 for ectopic expression constructs |
| CE260 | TTATGATGTCGGGCGGCCGCTTCGGAATAGGAACTTCAAGATCCCC | R1 for vanAB ectopic expression constructs |
| CE261 | CTTGAAGTTCCTATTCCGAAGCGGCCGCCCGACATCATAAC | Ptac promoter F |
| CE336 | GCTTAATTACCTCCTAATTGAATTCCTAGGC | Ptac promoter R |
| CE947 | CAATTAGGAGGTAATTAAGCATGTCTTCACAAGTTTCTATTCGCTTCGA | Ptac-fimL F for ectopic expression at vanAB locus |
| CE948 | TGTAGGCTGGAGCTGCTTCTTAGGCAACCGTTGATTGAAATTGGG | Ptac-fimL R for ectopic expression at vanAB locus |
| CE262 | ACTTTCTAGAGAATAGGAACTTATTACTTATATAATTCATCCATTCCACCCCCA | Ptac-mRuby3 R |
| CE263 | ATGAATTATATAAGTAATAAGTTCCTATTCTCTAGAAAGTATAGGAACTTCGAA | mRuby3 insertion into kanR cassette F2 |
| CE1509 | CAGGATGAGGATAGTTTCGCATGTCATCAGCGGTGGAGTG | ApramycinR gene F |
| CE1510 | ACTTCAAGATCCCCTTATTATGAGCTCAGCCAATCGACTG | ApramycinR gene R |
| CE1511 | GCGAAACTATCCTCATCCTGTCTCTTG | kanR gene replacement R1 |
| CE1512 | TAAGGGGATCTTGAAGTTCCTATTCCG | kanR gene replacement F2 |

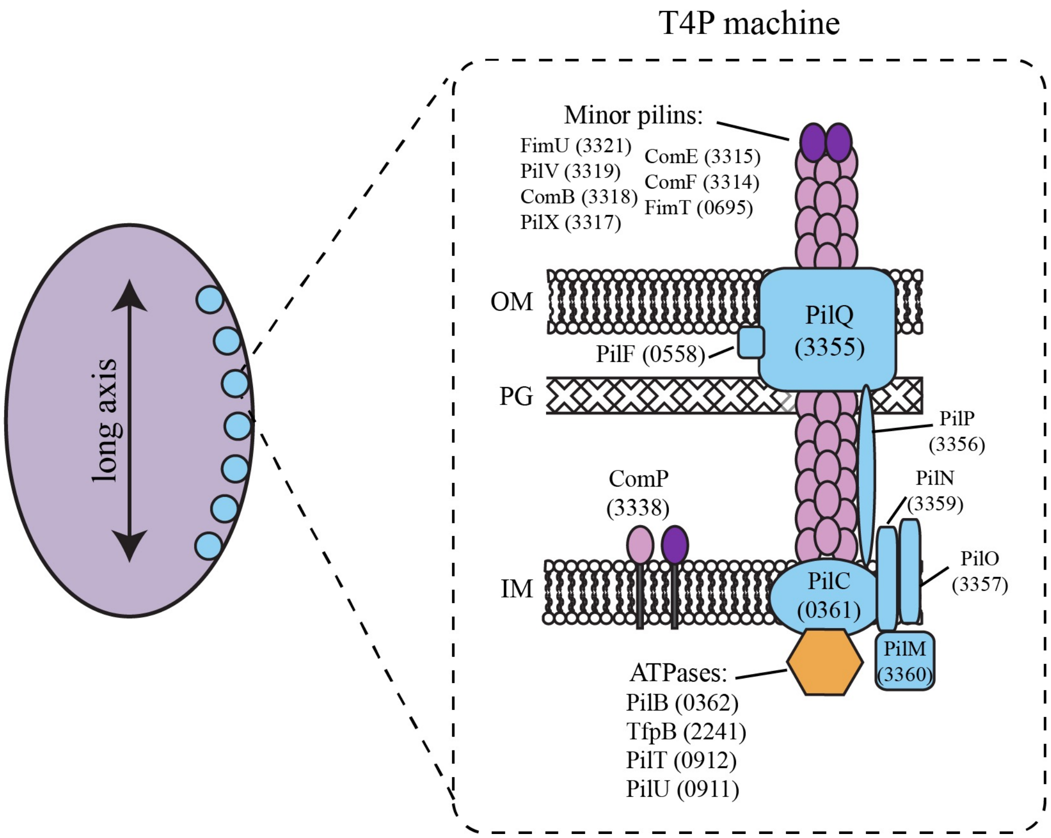


Supplementary Figure 1. *A. baylyi* T4P are produced in a line that is parallel to the long axis of the cell. Schematic of an *A. baylyi* cell (left) and the T4P machinery it produces (right).

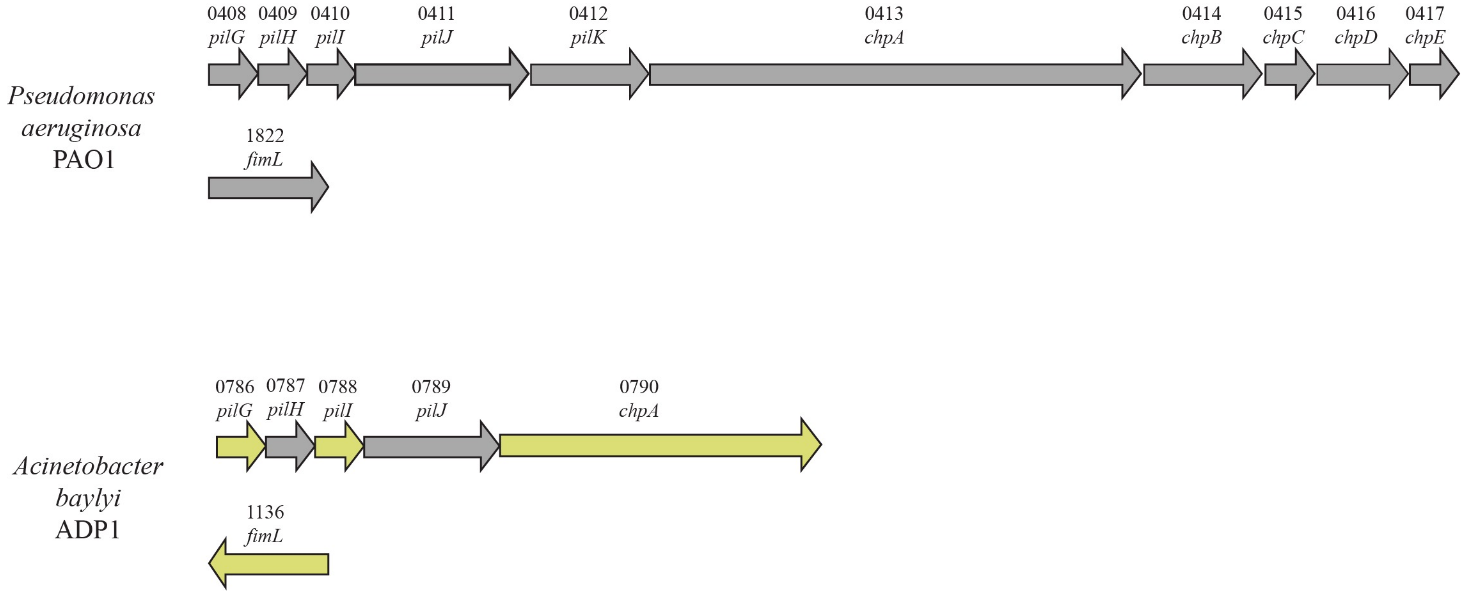


Supplementary Figure 2. The *A. baylyi* Pil-Chp pathway is missing several components found in *Pseudomonas aeruginosa*. Schematic of the Pil-Chp genes found in *P. aeruginosa* (top) and *A. baylyi* (bottom). Numbers above *P. aeruginosa* genes indicate PA numbers and numbers above *A. baylyi* genes indicate ACIAD gene numbers. Deletions of components colored gold cause dispersed T4P localization while deletion of components in gray have no effect on localization of T4P machines.

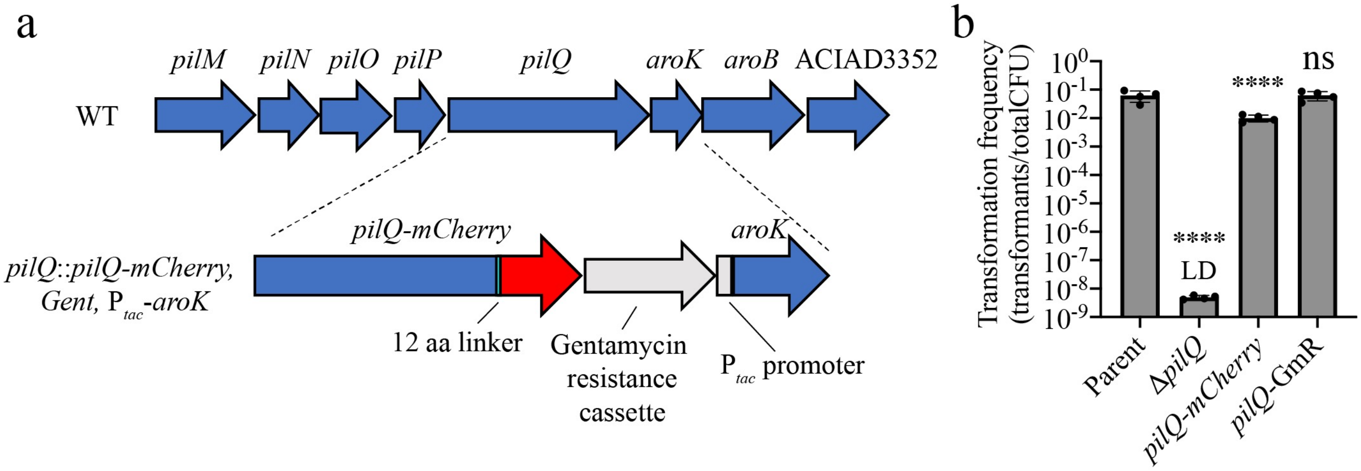


Supplementary Figure 3. Generation of a PilQ-mCherry fusion in *A. baylyi*. (a) Schematic of *pilQ-mCherry* gene locus. Attempts to make *pilQ-mCherry* by natural transformation without a selective marker were unsuccessful, and we thus added a gentamycin resistance cassette downstream of *pilQ*. Because *pilQ* is in an operon with the essential gene *aroK* immediately downstream, we added a constitutively active $P_{tac}$ promoter to drive *aroK* expression. (b) Natural transformation assays of indicated strains. The pilQ-GmR control strain contains the same GmR-$P_{tac}$ marker downstream of PilQ present in *pilQ-mCherry*, but PilQ is untagged in this background. Each data point represents a biological replicate (n = 4) and bar graphs indicate the mean ± SD. The transformation frequency of the Δ*pilQ* strain was below the limit of detection, indicated by LD. Statistical comparisons were made using One-Way ANOVA followed by Dunnett's multiple comparisons test comparing log-transformed values from mutant strains to the parent: ns, not significant; ****P < 0.0001. Exact measurements and P values are reported in the Source Data file. Genotypes of each strain used in each figure panel are outlined in Supplementary Table 1.

```
ADP1-fimV      ------------------------------------------------------------     0
PAO1-fimV      MVRLRTLVRAIAAASVLTSGMAHGLGLGEITLKSALNQPLDAEIELLEVRDLGSGEVIPS     60

ADP1-fimV      ------------------------------------------------------------     0
PAO1-fimV      LASPEEFSKAGVDRLYYLTDLKFTPVVKPNGKSVIRVTSSKPVQEPYLNFLVQVLWPNGR     120

ADP1-fimV      ------------------------------------------------------------     0
PAO1-fimV      LLREYTVLLDPPLYSPQAAASAPQAPVSAPRATGAPRAPQAPAPVRTTAPAGSDTYRTVS     180

ADP1-fimV      ------------------------------------------------------------     0
PAO1-fimV      NDTLWEIAQRNRTDRVSVPQAMLAFQELNPGAFVDGNINRLKSGQVLRIPTEQQMLERSP     240

ADP1-fimV      ------------------------------------------------------------     0
PAO1-fimV      REALSQVQAQNQSWRGSRNPAAGSAGARQLDATQRNAAGSAPSKVDATDNLRLVSGEGKA     300

ADP1-fimV      ------------------------------------------------------------     0
PAO1-fimV      SKGADKGGKGDSKAIADTLAVTKESLDSTRRENEELQSRMQDLQSQLDKLQKLIQLKDAQ     360

ADP1-fimV      ------------------------------------------------------------     0
PAO1-fimV      LAKLQGQLGAEGQGAAQPNAALPDASQPNAAAQAPAQPGTPAAAAPTPAPAGEAPAAPAQ     420

ADP1-fimV      --------------------------------------------MLYVIPFIILLVVAII     16
PAO1-fimV      PPVAPPPAPAAEKPPAPAVPAPAPVQAAEQPAPSFLDELLANP[illegible]WLAVIGGSALLALLVL     480
                                                            * **    **.: ::

ADP1-fimV      FII[illegible]QRRQNASSSSHAK--N-----NKNNEIKTSE---NDLSAAKT---PVTIDP-----     58
PAO1-fimV      LMI[illegible]SRRNAQKEKEEAQAFAADTGEEQEDALDLGKDGFDDLTLDEPEPQVAAVAPQVEKT     540
               ::* .**:  .....*:        :::: :. .:   :**:  :     .:: *

ADP1-fimV      -----NIHERIDHLIDIGHLSAAEALINQTLNRDPSRHDLYLYLLDLHLRQNDELGIKQL     113
PAO1-fimV      TAQTSDALGEADIYIAYGRFNQAAELLQNAIYDEPQRTDLRLKLMEVYAEMGDREGFARQ     600
                    :   . *  *  *::. *  *:::::  :*.* ** * *:::: . .*. *: :

ADP1-fimV      IQNLQQLQLHDVLNQIEGKYRNTHEQKEKYAKIIQQKQ---------------------Q     152
PAO1-fimV      ENELR---------EIGGAQPQVEQLKSRYPAMVAVAAVAGLAGAKLAQDELDSFSLDDL     651
                ::*:         :* *   :..: *.:*  ::

ADP1-fimV      NIQDITRTASPTATA-----AIKVETTHTNPAPVQKHIIEEHQSL---EFNPKPIAPVNK     204
PAO1-fimV      SLDDSGHAAKPDAAGQDLDDAFDLSLDDLGGDDVQADLKSDSGALDDLTLDSDL--DL-A     708
               .::*  ::*.* *:.     *:.:.  . .   ** .: .:  :*    :: .    :

ADP1-fimV      SEKSNTVPHQDISFVLEMADQITV--SPKKNEAA-AEVKVENQTRK---------LAASP     252
PAO1-fimV      ASTPADKPVDDLDFGLDFAELAETPSQPKHDDLGDFSLDLDAPEDKLSDDDFLLSLNDEV     768
               :..    * :*:.* *::*:   .  .**::: .  .:.::    *         *  .

ADP1-fimV      PQALNADQEASHLAQPKVVEPRIDTPP------VHTPVPPPTLTRQ-DTPPITL---KQH     302
PAO1-fimV      PAAAPADNEF-TLDTEAAEEPALSLPDDFDLSLADEPTEPAAPEKGEDSFAAQLDEVSAQ     827
               * *  **:*   *    . ** :. *       .. *. * :  :  *:    *   . :

ADP1-fimV      LPPVAESL----------QPQPAQI-TVDHDPLIQAFPELANLDETQL[illegible]LELAEQYIELG     351
PAO1-fimV      LDELASNLDEPKSATPSFSAEDAAVASALDGDADDDFDFLSGADEAAT[illegible]LDLARAYIDMG     887
               *  :*..*          . : * : :. ..   : *  *:. **:   *:**. **::*

ADP1-fimV      AYASAQILLNQNESKFSAQQLELSKKLLNRMAS*     384
PAO1-fimV      DSEGARDILDEVLAEGNDSQQAEARELLERLA*-     919
                  .*: :*::  :: . .*   :::**:*:*
```

Supplementary Figure 4. FimV in *A. baylyi* is truncated and shares similarity to the C-terminus of *P. aeruginosa* FimV. ClustalW[4] alignment of full length FimV proteins from either *A. baylyi* ADP1 (ACIAD0477) or *P. aeruginosa* PAO1 (PA3115). Blue boxes indicate a conserved, predicted transmembrane domain, and the red boxes indicate the conserved, predicted C-terminal 40-aa FimV domain.

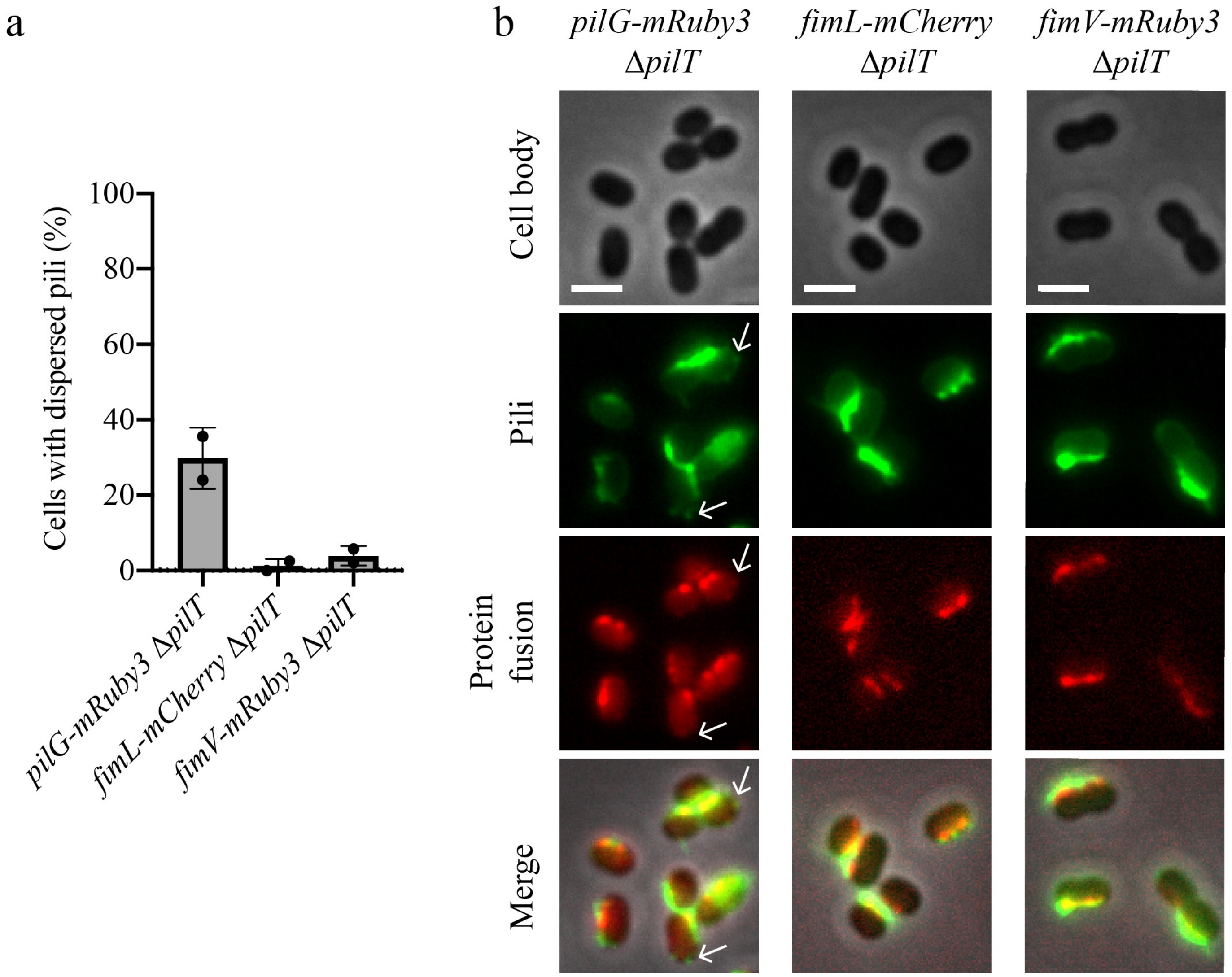


Supplementary Figure 5. Functionality of Pil-Chp protein fusion strains. (a) Quantification of the percentage of cells in a population with dispersed T4P. Cells with dispersed T4P were defined as cells that had T4P on multiple sides of the cell body. Each data point represents an independent, biological replicate (n = 2) and bar graphs indicate the mean ± SD. For each biological replicate, a minimum of 30 total cells were assessed. (b) Representative images of indicated strains with fluorescently labeled T4P and background fluorescence subtracted. White arrows indicate dispersed T4P in the PilG-mRuby3 strain indicating partial functionality. Scale bars, 2 µm. Genotypes of each strain used in each figure panel are outlined in Supplementary Table 1.

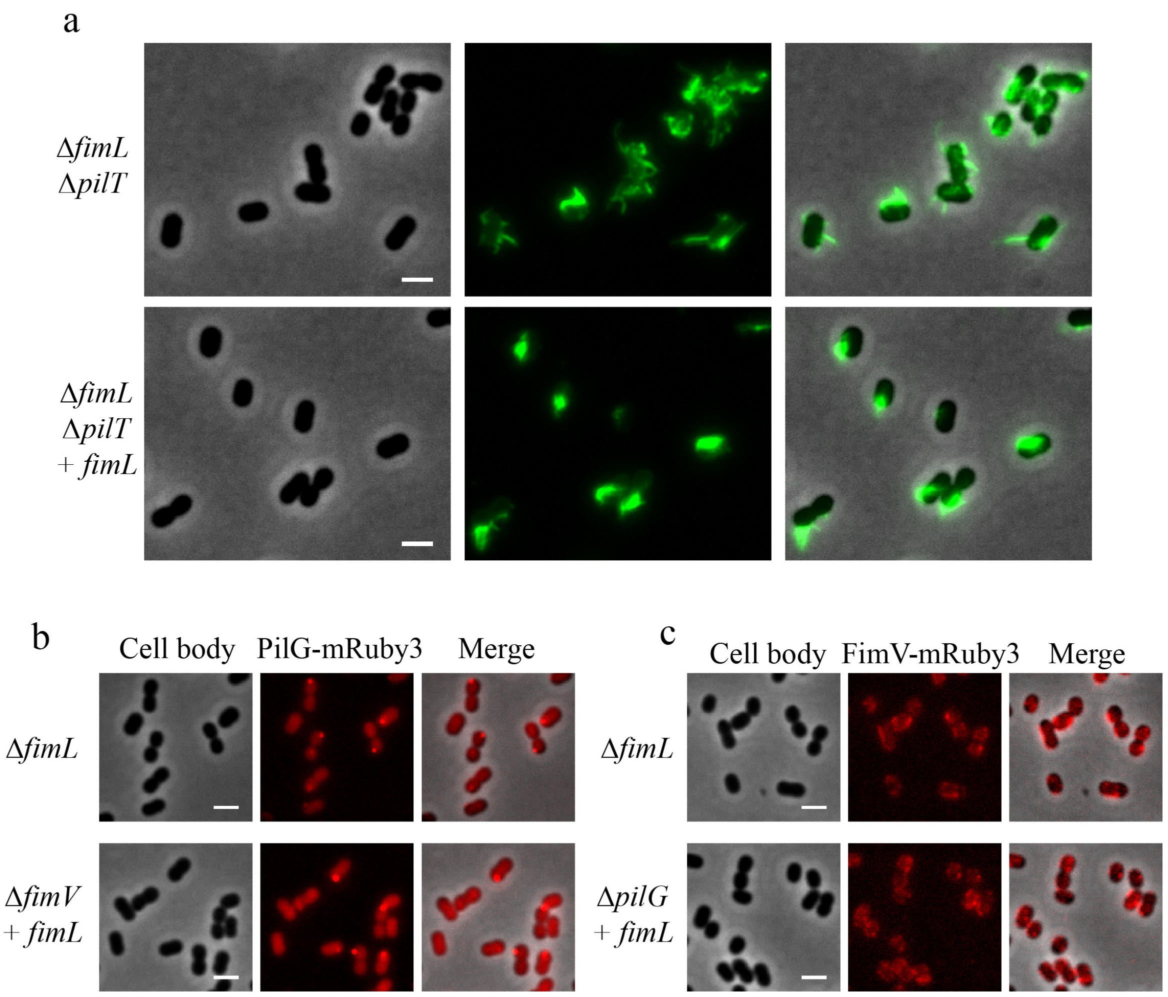


Supplementary Figure 6. Ectopic expression of *fimL* complements a *fimL* deletion but not localization patterns of Pil-Chp components. (a) Representative images of indicated strains with fluorescently labeled T4P and background fluorescence subtracted. (b) Representative images of indicated PilG-mRuby3 strains with background fluorescence subtracted. (c) Representative images of indicated FimV-mRuby3 strains with background fluorescence subtracted. Scale bars, 2 µm. Genotypes of each strain used in each figure panel are outlined in Supplementary Table 1.

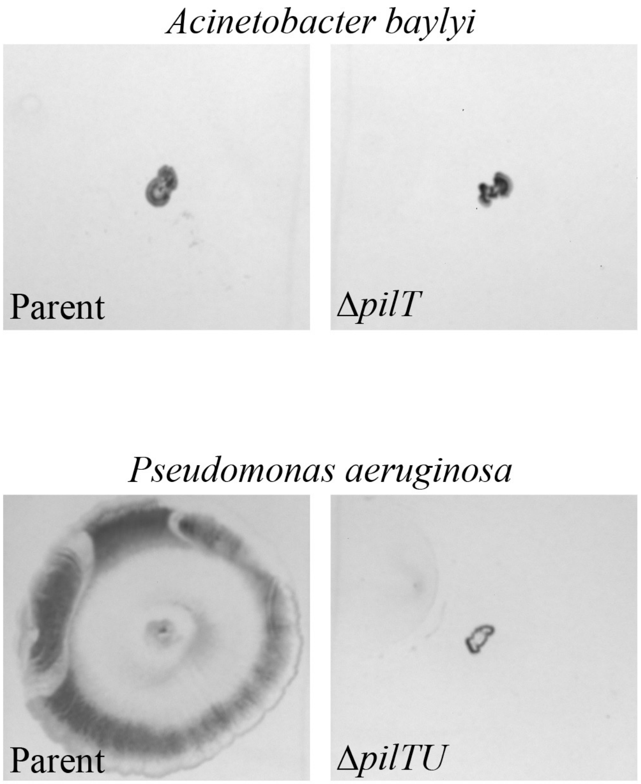


Supplementary Figure 7. *A. baylyi* does not exhibit classical twitching motility by standard laboratory assays. Images show twitch assays in which cells were inoculated between 1.5% agar medium and the bottom of a petri plate comparing parent strains (left) from either *A. baylyi* (top) or *P. aeruginosa* (bottom). Δ*pilT* mutants, which are defective in T4P motility, are used as controls (right). Genotypes of each strain used in each figure panel are outlined in Supplementary Table 1. Source data are provided in a Source Data file.

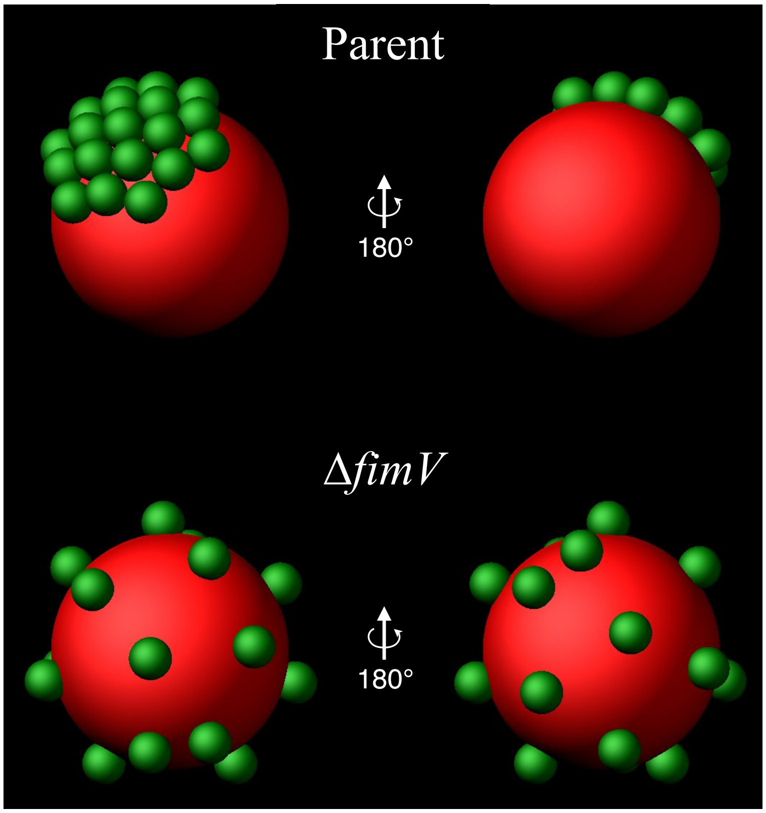


Supplementary Figure 8. Representations of the modeled cellular objects used in molecular dynamics simulations. Green spheres represent adhesins and red spheres represent cells.

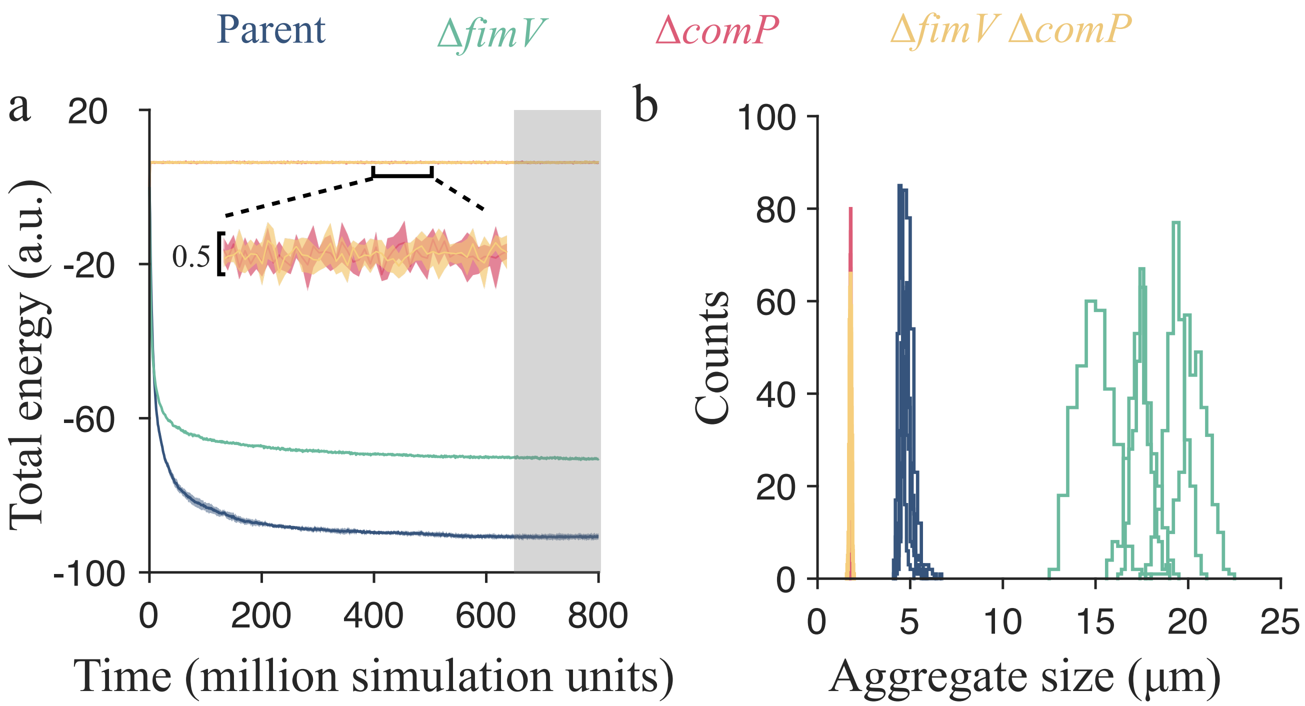


Supplementary Figure 9. Data collection and aggregate size quantification in simulations. Five replicates of simulations were run for each of the four simulated strains: Δ*fimV*Δ*comP* (yellow), Δ*comP* (red), Δ*fimV* (green), and parent (blue). (a) Total energy of the simulation system versus time. For each strain, the solid curve with shaded error band represents mean ± SD of the five replicates. Shaded gray area indicates the period in which the system was considered to be equilibrated and data were collected for further quantification. Inset: blow-up of a segment of the time series for Δ*fimV*Δ*comP* and Δ*comP*. (b) Size distributions of the simulated cell aggregates. A histogram is shown for each simulation replicate; each replicate was stopped at 300 different times in the equilibrium period in a, compressed, and analyzed for aggregate size (see Methods).

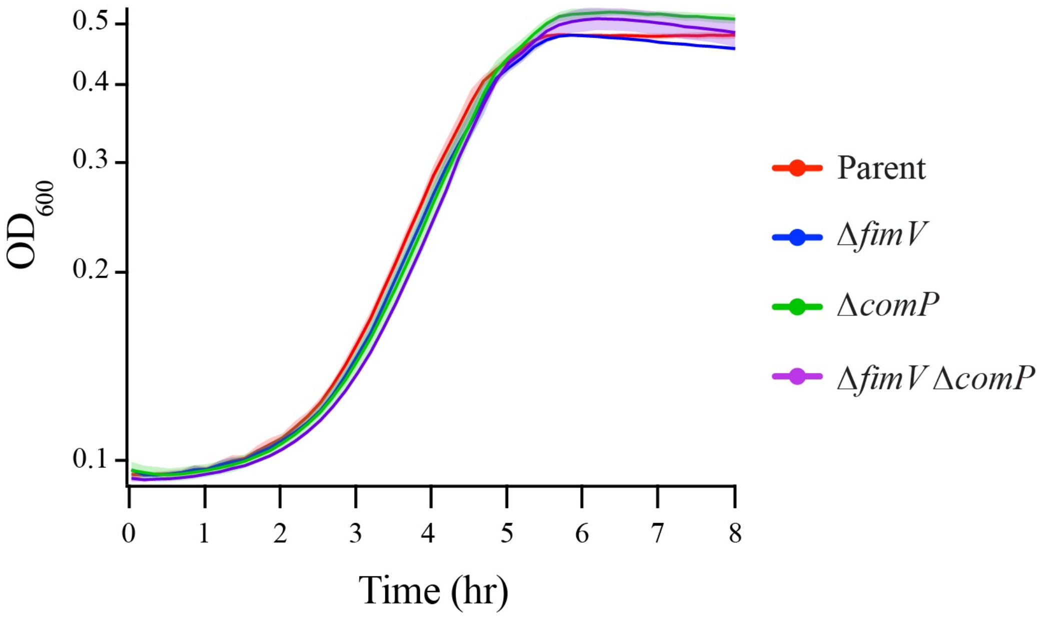


Supplementary Figure 10. *fimV* and *comP* deletion strains exhibit wildtype growth rates. Growth curves from three biological replicates for indicated strains. Lines and shaded regions indicate the mean ± SD. Genotypes of each strain used in each figure panel are outlined in Supplementary Table 1.
